# Bio-Based Epoxy Vitrimers with Excellent Properties of Self-Healing, Recyclability, and Welding

**DOI:** 10.3390/polym16152113

**Published:** 2024-07-24

**Authors:** Jianrong Xia, Shuyun Li, Renjin Gao, Yuchi Zhang, Liwei Wang, Yuansong Ye, Changlin Cao, Hanyu Xue

**Affiliations:** 1 Fujian Engineering and Research Center of New Chinese Lacquer Materials, Fujian Key Laboratory of Functional Marine Sensing Materials, Minjiang University, Fuzhou 350108, China; jrxia@mju.edu.cn (J.X.); 17623111224@163.com (S.L.); 1248@mju.edu.cn (R.G.); zyc@mju.edu.cn (Y.Z.); wlw@mju.edu.cn (L.W.); yeyuansong@mju.edu.cn (Y.Y.); 2 College of Environmental and Resource Sciences, Fujian Normal University, Fuzhou 350108, China

**Keywords:** bio−based epoxy vitrimer, transesterification, recycling, cardanol, vanillyl alcohol

## Abstract

The development of more recyclable materials is a key requirement for a transition towards a more circular economy. Thanks to exchange reactions, vitrimer, an attractive alternative for recyclable materials, is an innovative class of polymers that is able to change its topology without decreasing its connectivity. In this work, a bisphenol compound (VP) was prepared from saturated cardanol, i.e., 3−pentadecylphenol and vanillyl alcohol. Then, VP was epoxidized to obtain epoxide (VPGE). Finally, VPGE and citric acid (CA) were polymerized in the presence of catalyst TBD to prepare a fully bio−based vitrimer based on transesterification. The results from differential scanning calorimetry (DSC) showed that the VPGE/CA system could be crosslinked at around 163 °C. The cardanol−derived vitrimers had good network rearrangement properties. Meanwhile, because of the dynamic structural elements in the network, the material was endowed with excellent self−healing, welding, and recyclability.

## 1. Introduction

With many advantages, such as excellent chemical resistance, high mechanical strength, excellent electrical performance, corrosion resistance, aging resistance, good dimensional stability, and high thermal stability, traditional thermosetting materials with a permanent crosslinked network structure, such as epoxy resin and rubber, have a wide range of applications in many fields, for example, the adhesives and coating industry, but their permanent crosslinked network structure limits their reprocessability and recyclability. Thermoplastics can be reprocessed and molded because of their linear structure, which has good reprocessing properties and recyclability [1,2,3,4,5,6]. The rapid development of dynamic covalent chemistry in polymer chemistry has dramatically diminished the gap between thermoplastics and thermosets, making it possible for the recyclable use of thermosetting resins. The concept of “vitrimer” was proposed in 2011, which combines the advantages of thermoset materials and thermoplastic materials, with good mechanical strength at lower temperatures and similar fluidity to thermoplastic materials at higher temperatures, giving them reprocessability and recyclability. Since the advent of vitrimers, more and more researchers have begun to focus on this field [7,8,9,10,11,12,13]. At first, researchers used bisphenol A glycidyl ether from petroleum resources, but with the gradual increase in awareness of environmental protection, bio−based vitrimers have become a current research hotspot in order to reduce environmental pressure [14,15,16]. Bio−based epoxy vitrimers prepared from renewable raw materials such as vegetable oils [17,18,19], natural rubber [20,21,22,23,24], vanillin [25,26,27], etc., have been widely reported. Altuna and co−workers prepared a fully biotransester−based epoxy vitrimer based on epoxy soybean oil and bio−based citric acid under catalyst−free conditions. Due to the low crosslinking density of this network and the large number of hydrogen bonds in the network, transesterification can be performed without catalysts [28]. Yang and co−workers used bio−based rosin triacid as the curing agent to cure epoxidized soybean oil (ESO) and added a small amount of zinc salt catalyst to prepare a transesterification−based bio−based epoxy vitrimer, which has excellent tensile properties, good self−healing properties, and excellent shape memory functions [29]. Wu and co−workers synthesized fully bio−based vitrimers with self−healing, recyclable, and shape memory features using epoxy soybean oil and glycyrrhizic acid [30]. Zhao and co−workers synthesized vitrimers with tunable properties using vanillol and 4−methylcatechol to synthesize substances with three phenolic hydroxyl groups [31].

**Scheme 1:**
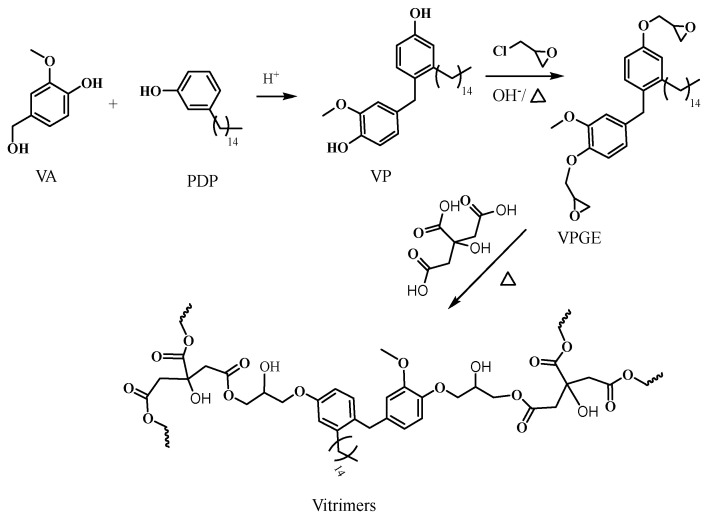
Schematic synthesis of VP, VPGE, and the vitrimers.

However, there are few reports on the vitrimers of natural phenols, and in this paper, we chose to synthesize a substance with two phenolic hydroxyl groups with cardanol and vanillyl alcohol and then solidified it with citric acid to prepare fully bio−based vitrimers with self−healing and reprocessing features. Cardanol is obtained from natural cashew nut shell oil as a raw material and is a green and environmentally friendly industrial raw material [32,33,34]. Vanillyl alcohol is an aromatic alcohol extracted from vanilla and gastrodia. Many kinds of fruits and vegetables, especially citrus fruits, contain high levels of citric acid, especially lemons and limes, which contain a lot of citric acid, which can reach 8% after drying. Since cardanol has only one phenolic hydroxyl group, inspired by Zhao and co−workers [31], as seen in Scheme 1, we selected 3−pentadecylphenol and vanillyl alcohol to design and synthesize a bisphenol compound (VP), and the VP was epoxidized to obtain epoxide (VPGE) and cured with citric acid in the presence of catalyst TBD. Since VP has only two phenolic hydroxyl groups, in order to enable the vitrimers to form a crosslinking network, we chose a substance with at least three carboxyl groups for curing when selecting an acid curing agent, and we chose citric acid for curing due to its low price. This work expands the applications of low−priced cashew phenols in vitrimer materials and provides a novel approach to prepare bio−based vitrimers with desirable properties. The structures of cardanol−derived VP and VPGE were characterized by nuclear magnetic resonance. The curing reactivity of VPGE was measured by differential scanning calorimetry (DSC). Additionally, the network rearrangement properties of cardanol−derived vitrimers were investigated by dynamic mechanical analysis (DMA).

## 2. Materials and Methods

### 2.1. Materials

Vanillyl alcohol (VA, 98%), 1,5,7−triazabicyclo[4.4.0]dec−5−ene (TBD, 98%), zinc(II) acetylacetonate (>96%), epichlorohydrin (ECH, AR), and tetrabutylammonium bromide (AR) were purchased from Aladdin. Sodium sulfate was purchased from Sinopharm Chemical Reagent Co., Ltd, Shanghai, China. 3−pentadecylphenol (PDP, >90%) was purchased from Macklin. All materials were used directly as purchased without further purifying.

### 2.2. Preparation of VP

VA (12.59 g, 80 mmol) and PDP (40.64 g, 120 mmol) were dissolved in 100 mL of absolute ethanol in a 250 mL there−necked flask. Under the ice water bath, 22.8 g of concentrated sulfuric acid was dissolved in 20 mL of absolute ethanol. The concentrated sulfuric acid anhydrous ethanol mixture solution was dropwise added slowly to the three−neck flask by a constant pressure drip funnel while stirred. After the addition of sulfuric acid, the temperature was increased to room temperature, and the mixture was stirred at room temperature for 2 h. After the completion of the reaction, the product was extracted by 100 mL of ethyl acetate and washed with saturated saline thrice. The extract was dried with Na_2_SO_4_ for 24 h and concentrated using a rotary evaporator. Purification of the product using silica gel chromatography (ethyl acetate/n−hexane, 1:3 to 1:4) obtained VP as a yellowish solid (25.34 g, 72% yield). ^1^H NMR (400 MHz, DMSO−d_6_) δ 9.04 (s, 1H), 8.67 (s, 1H), 6.95–6.27 (m, 7H), 3.73 (s, 2H), 3.68 (s, 3H), 2.41 (d, J = 7.9 Hz, 2H), 1.36 (s, 2H), 1.23 (s, 24H), 0.85 (s, 3H). ^13^C NMR (400 MHz, DMSO−d_6_) δ 156.00, 147.79, 144.95, 142.01, 132.74, 131.31, 129.50, 121.00, 116.25, 115.70, 113.15, 113.01, 55.94, 37.40, 31.79, 30.86, 29.54, 29.21, 22.59, 14.41.

### 2.3. Preparation of VPGE

VP (4.41 g, 10 mmol) and TBAB (0.26 g, 0.85 mmol) were dissolved in ECH in a 100 mL three−necked flask with a thermometer and a condenser−west tube. The mixture was heated at 60 °C for 3.5 h. Then, 4 g of 20% *w*/*w* NaOH solution was dropwise added into the mixture. The mixture was kept at 60 °C for another 1.5 h. The product was extracted by 20 mL ethyl acetate and washed with saturated saline thrice. The extract was dried with Na_2_SO_4_ for 24 h and concentrated using a rotary evaporator, which gave a yellowish solid (4.12 g, 75% yield). ^1^H NMR (400 MHz, DMSO−d_6_) δ 7.01 (d, J = 8.2 Hz, 1H), 6.83 (d, J = 8.2 Hz, 1H), 6.73 (ddd, J = 14.2, 7.6, 2.3 Hz, 3H), 6.52 (dd, J = 8.2, 2.0 Hz, 1H), 4.23 (ddd, J = 25.5, 11.3, 2.8 Hz, 2H), 3.83 (s, 2H), 3.77–3.72 (m, 2H), 3.70 (s, 3H), 3.29 (dq, J = 6.8, 2.1 Hz, 2H), 2.84–2.79 (m, 2H), 2.67 (ddd, J = 13.5, 5.1, 2.7 Hz, 2H). 

### 2.4. Preparation of VPGE−CA Vitrimers

CA was dissolved in absolute ethanol and dissolved with ultrasound assistance until CA was completely dissolved. VPGE was added to the CA absolute ethanol solution at 120 °C with a magnetic stirrer. TBD (5% molar ratio to carboxylic acid) was added into the mixture and stirred until it was well combined. The ratios of the epoxy groups and –COOH groups were set to R, and vitrimers samples were prepared at R = 1.0, 0.8, and 0.6, respectively. The composition of various samples is listed in Table 1. The mixture was poured quickly into a Teflon mold which was preheated at 120 °C for 10 min. Then, the mold was put into a vacuum−pressure desiccator and degassed under vacuum to remove entrapped air for about 20 min and was then cured at 100 °C for 3 h, 140 °C for 4 h, and 170 °C for 4 h.

### 2.5. Characterizations

Attenuated total reflectance Fourier transform infrared (ATR−FTIR) spectra were recorded using a Thermo Nicolet iS5 spectrometer (Thermofisher Scientific, Waltham, MA, USA). A total of 32 scans were conducted with a resolution of 4 cm^−1^. ^1^H NMR and ^13^C NMR spectra were performed in DMSO−d_6_ by a 400 MHz Bruker−BioSpin AVANCE III HD spectrometer (Bruker Biospin, Fällanden, Switzerland) with tetramethylsilane (TMS) as the internal reference. 

The sample curing process and glass transition temperature were tested using a UV difference scanning calorimeter (DSC2500, TA Instruments, New Castle, DE, USA). The uncured sample was scanned from 30 °C to 250 °C at a heating rate of 10 °C/min nitrogen atmosphere. When testing the glass transition temperature of the sample, we weighed about 10 mg of the sample into an aluminum crucible and the test process was first heated from 30 °C to 250 °C at a heating rate of 10 °C/min, kept warm for 5 min, and then cooled down from 250 °C to −70 °C at a heating rate of 10 °C/min.

Dynamic thermomechanical analysis was measured using the TA−Q800 (TA Instruments, New Castle, DE, USA) instrument with a sample dimension of 30 mm × 5 mm × 1 mm. The test temperature range was −50–200 °C at a heating rate of 5 °C/min. The frequency was 1 Hz, and the load force was 0.01 N.

Stress relaxation was measured using the TA−Q800 instrument (TA Instruments, New Castle, DE, USA) with a sample dimension of 30 mm × 5 mm × 1 mm. The temperature was heated to the measured temperature and insulated for 2 min, the static stress was 0.001 N, and then a constant strain of 1% was applied to record the change in relaxation modulus with time.

The thermal stability test was carried out by NETZSCH TG 209F3 (NETZSCH Waldkraiburg, Germany) with the sample (~10 mg) in an alumina ceramic crucible. The temperature heated from 30 °C to 600 °C at a rate of 10 °C/min in a nitrogen atmosphere.

Solvent Resistance Test: The VPGE−CA vitrimers sample (~20 mg) was placed in a 5 mL vial. Its original mass was recorded as m_a_. A total of 2 mL of n−hexane, toluene, ethyl acetate, chloroform, tetrahydrofuran, acetone, N,N−dimethylformamide, methanol, water, and other solvents of different polarities was added, respectively, and soaked at room temperature for 24 h. Then, the sample was removed, and the surface solvent was wiped with filter paper; the mass was recorded as m_b_. The sample was placed in an oven at 150 °C for 2 h and the mass m_c_ was recorded. The swelling ratio and gel content of the sample in different solvents were calculated as follows:

(1) $$swelling\;ratio\left(\%\right)=\frac{m_{b}-m_{a}}{m_{a}}\times 100\%\;$$

(2) $$gel\;content\left(\%\right)=\frac{m_{c}}{m_{a}}\times 100\%\;$$

Tensile performance was tested using a universal testing machine at a tensile rate of 10 mm/min with a sample dimension of 50 mm × 10 mm × 1 mm.

Repairing Property Test: A scalpel was used to create scratches on the sample, and then the sample was placed in a 180 °C oven for repair experiments. The crack was observed with a light microscope at 0 min, 5 min, and 60 min, respectively.

Chemical Degradation Test: The sample (~20 mg) was soaked in a vial containing 5 mL of ethylene glycol, and the initial mass of the sample was recorded as m_a_. The vial was placed in an oven at 180 °C, and the sample was taken out at the same interval, oven−dried to constant weight, and the mass of the sample was weighed as m_b_. The relative weight was calculated as follows:

(3) $$relative\;weight\left(\%\right)=\frac{m_{b}}{m_{a}}\times 100\%$$

The physical recovery properties were studied using a universal testing machine. The sample was cut with a scalpel and evenly spread on the flat vulcanizer mold. A new sample was formed by vulcanizing at a temperature of 200 °C for 120 min on a pressure vulcanizer at 200 kg/cm^2^.

## 3. Results and Discussion

### 3.1. Structure Characterization of VP and VPGE

**Figure 1:**
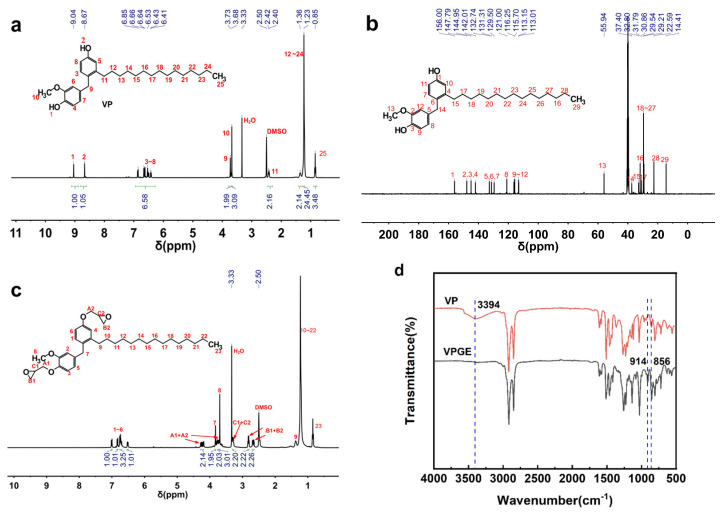
(**a**) ^1^H NMR spectra of VP, (**b**) ^13^C NMR spectra of VP, (**c**) ^1^H NMR spectrum of VPGE, and (**d**) FTIR spectra of VP and VPGE.

The structure of VP and VPGE was confirmed by ^1^H NMR, ^13^C NMR, and Fourier transform infrared (FTIR) spectra. VP containing two phenolic hydroxyl groups was synthesized through the condensation of VA and PDP. As seen in Figure 1a,b, the methylene bridge of VP was observed at 3.73 ppm in the ^1^H NMR spectra and at 37.4 ppm in the ^13^C NMR, which suggested successful linkage between VA and PDP [31]. As seen in Figure 1c, the disappearance of the peaks of the two phenolic hydroxyl groups at 9.04 and 8.67 ppm indicates that the reaction was complete. As seen in Figure 1d, the appearance of epoxy group peaks at 912 cm^−1^ and 854 cm^−1^ and the disappearance of the hydroxyl groups peak at 3394 cm^−1^ proved the successful preparation of VPGE.

### 3.2. Curing and Structure Characterization of VPGE−CA Networks

**Figure 2:**
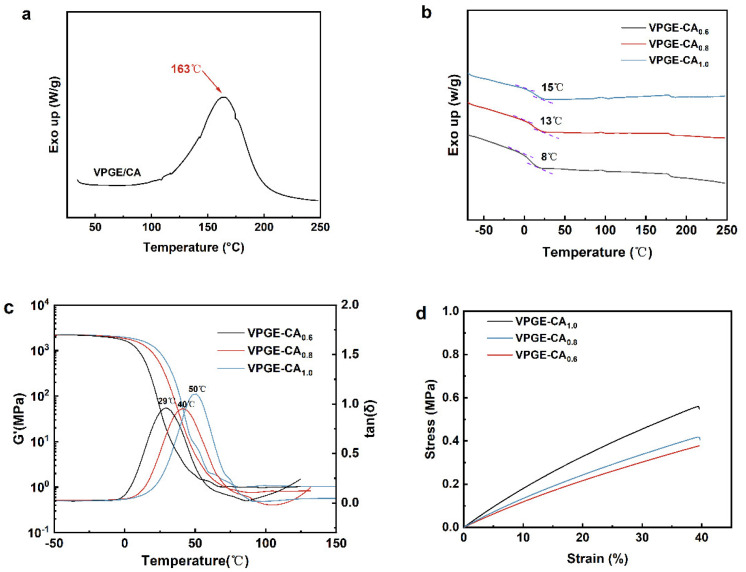

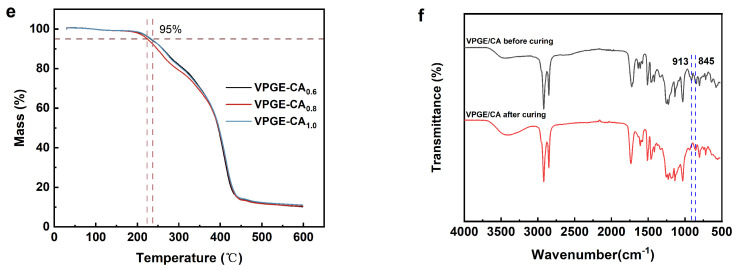
(**a**) DSC curves of VPGE−CA (R = 1.0, TBD 5 mol%), (**b**) DSC curves of VPGE−CA (R = 0.6, 0.8, 1.0, TBD 5 mol%), (**c**) storage modulus (left) and tan δ (right) of VPGE−CA networks (R = 0.6, 0.8, 1.0; TBD 5 mol %), (**d**) strain–stress curves of VPGE−CA networks (R = 0.6, 0.8, 1.0; TBD 5 mol %), (**e**) TGA curves of VPGE−CA networks (R = 0.6, 0.8, 1.0; TBD 5 mol %), and (**f**) FTIR spectra of VP−CA before curing and after curing.

VPGE contains two epoxy groups, and citric acid was selected to cure VPGE to prepare vitrimers. The curing reactions of VPGE and CA were performed in the presence of TBD (5% molar ratio to carboxylic acid), which served as a catalyst for the transesterification reactions. In order to investigate the effect of the ratio of different epoxy groups to –COOH groups on the properties of the prepared polymers, the ratios of three epoxy groups to –COOH groups were designed, which were denoted as R. The stoichiometric ratios (R = epoxy/carboxylic acid) were set up as 0.6, 0.8, and 1.0. The study of curing behavior was performed by DSC to establish the temperature of the curing process. As seen in Figure 2a, the result shows that the VPGE/CA system needs to be cured and crosslinked under high−temperature conditions, and the exothermic peak is around 163 °C, so we set the curing program of 100 °C/3 h + 140 °C/4 h + 170 °C/4 h for curing in the oven. As seen in Figure 2f, the peak at 3500 cm^−^^1^ was attributed to the hydroxyl group of the vitrimers, and the characteristic peak of epoxy group absorption is 913 cm^−^^1^. When the system was fully cured, the characteristic peak of 913 cm^−^^1^ disappeared. 

To investigate the network crosslinking and thermal properties of the samples, we tested the glass transition temperature of the sample with DSC, and the results are shown in Figure 2b, where the glass transition temperatures of the three systems were 8 °C, 13 °C, and 15 °C, respectively. With the increase in R, the glass transition temperature increased gradually, indicating that the crosslinking density of the network was the highest under the ratio of R = 1. This might be due to the fact that at R = 1, one mole of epoxy groups could be combined for one mole of carboxyl groups, which ensured the maximum crosslinking density. At the ratio of R = 0.6 and R = 0.8, not every epoxy group could be crosslinked with a carboxyl group, so there would be a free hydroxyl group, which reduces the crosslinking density. A similar trend was observed at around 95 °C and 175 °C, probably owing to the transition of the motion state of the main chain skeleton of the vitrimers.

The viscoelastic characterization of VPGE−CA by DMA was introduced to further investigate the crosslinking density of the network. As seen in Figure 2c, the peak temperature of tan δ in DMA could be regarded as the glass transition temperature of the material, and for the three systems of VPGE−CA, their Tα was 29 °C, 40 °C, and 50 °C, respectively. The trend in Tα was consistent with the results of DSC characterization. Therefore, it could be found that when R = 1.0, the crosslinking density of the VPGE−CA network was the largest, so the Tα was the highest. It should be noted here that the glass transition temperature values vary greatly depending on the measurement method.

At the same time, the mechanical properties and thermal stability of the material were characterized. As seen in Figure 2d, there was little difference in the strain at break between the three proportions of the VPGE−CA networks, and the strain at break was more than 40%, which might be due to the fact that the long side chain of 3−pentadecylphenol provided flexibility to the network, making the elongation at break almost identical. The 5% weight loss temperature in all three systems was more than 200 °C, as shown in Figure 2e, demonstrating the VPGE−CA network’s excellent thermal stability.

### 3.3. Network Rearrangement Properties

**Figure 3:**
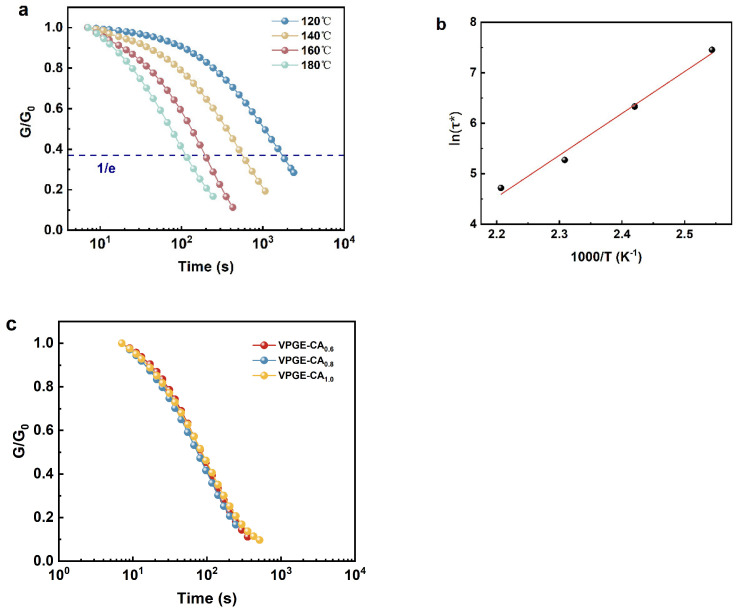
(**a**) Stress relaxation curves of VPGE−CA (R = 1.0, TBD, 5% molar ratio to carboxylic acid) networks at different temperatures (120 °C, 140 °C, 160 °C, 180 °C), (**b**) Arrhenius curve fitted by relaxation time (τ^∗^) obtained by (**a**,**c**) stress relaxation curves of VPGE−CA (R = 1.0, R = 0.8, R = 0.6, TBD, 5% molar ratio to carboxylic acid) networks at 180 °C.

The ester bond in transesterification was a type of dynamic covalent bond, which allows for a transesterification reaction at high temperatures. As a result, the viscosity of the vitrimers showed a slow gradual process with the increase in temperature, and the transition process was controlled by the kinetics of the exchange reaction, which indicated the viscosity change law in accordance with the Arrhenius equation. The relaxation velocity in stress relaxation was usually used to characterize the reaction rate of dynamic bonds in a network [35,36]. Therefore, one of the most crucial characteristics used to describe the network’s rearrangement behavior was stress relaxation. The time it takes for the material stress (or modulus) to relax to 1/e (G/G_0_ = 1/e) is known as the relaxation time (τ^∗^). As seen in Figure 3a, the evolution of the relaxation modulus of the VPGE−CA vitrimer network (R = 1.0, TBD 5 mol%) was found at 120 °C, 140° C, 160 °C, and 180 °C, respectively. According to the stress relaxation curve obtained by the stress relaxation test, the relaxation time of the material was 1735 s at 120 °C, 556 s at 140 °C, 196 s at 160 °C, and 112 s at 180 °C. With the increase in temperature, the relaxation time was shortened from 1735 s at 120 °C to 112 s at 180 °C. The reason for this phenomenon is that the increase in temperature accelerated the transesterification reaction rate in the network and thus accelerated the rearrangement rate of the network, so that the relaxation time was significantly shortened. As seen in Figure 3b, we performed the Arrhenius curve for the relaxation time in Figure 3a. The Arrhenius equation is shown in Equation (4).

(4) $$\ln\tau^{*}=\frac{Ea}{RT}-lnA$$

where τ^∗^ is the relaxation time, Ea is the activation energy of the bond exchange, T is the thermodynamic temperature at relaxation, and R is the gas molar constant. The linear equation fitted by the Arrhenius equation was Equation (5). E_a_ was 77 kJ/mol as calculated. The linear fitting correlation coefficient was 0.9916, and the closer the linear fitting correlation coefficient was to 1, the smaller the fitting deviation.

(5) $$y=9.29132x-13.70497\left(R²=0.9916\right)$$

In addition, the stress relaxation behavior of the samples of different proportions (R = 1.0, R = 0.8, R = 0.6, TBD, 5% molar ratio to carboxylic acid) at 180 °C was also studied. As seen in Figure 3c, the results showed that the stress relaxation behavior was almost the same under different ratios of epoxy and carboxylic acids. In 2011, Leibler and co−worker [7] proposed a vitreous topological freezing transition temperature feature called T_v_. This characteristic temperature is defined as the temperature at which the transition from solid to liquid occurs at a viscosity of η = 10^12^, i.e., above this temperature, the network topology can be rearranged by dynamic exchange. According to the Maxwell model, we could obtain T_v_ = 57 °C for the materials.

### 3.4. Repairing, Welding, and Shape−Changing Properties

**Figure 4:**
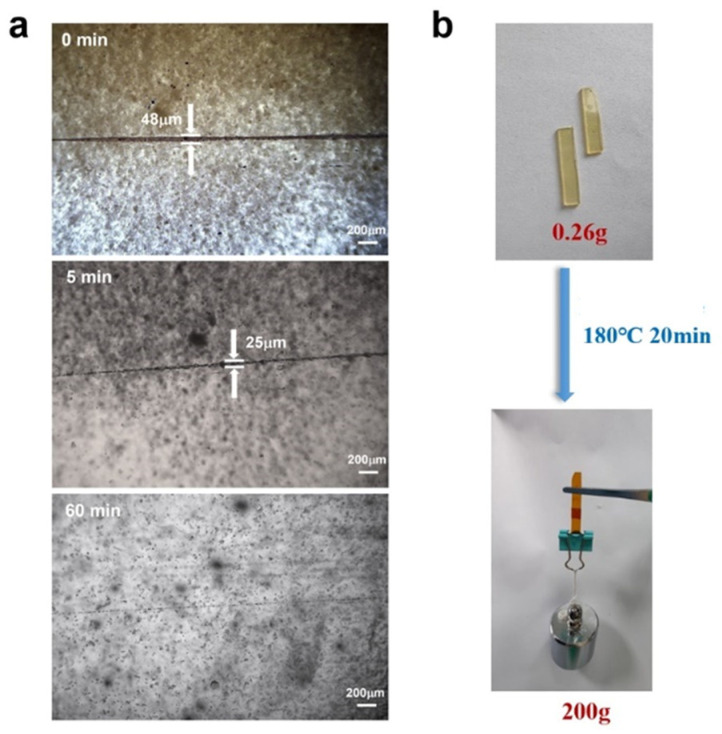
(**a**) Optical microscope images of the crack reparation of (R = 1.0, TBD 5 mol%) at 180 °C and (**b**) welding test of (R = 1.0, TBD 5 mol%).

Through stress relaxation experiments, we determined that VPGE−CA_1.0_ is a dynamic network structure with a transesterification reaction. Therefore, VPGE−CA_1.0_ vitrimers could be made to have the properties of repair, shape memory, and so on through network rearrangement. Scratches on the surface of the material were created by a scalpel, and after a period of thermal repair experiments, we could observe the repairing property by observing the changes in the cracks through an optical microscope. As seen in Figure 4a, after 5 min, the crack decreased significantly from 48 microns to 25 microns. The crack was almost gone after 60 min. The results demonstrated the outstanding repair ability of the VPGE−CA_1.0_ network.

Two straight strip samples were partially laminated under pressure and heated in an oven at 180 °C for 20 min. Due to the transesterification reaction that occurs at the interface of the two laminated parts at high temperatures, it is possible to join the two straight strips together until the bonding points are integrated with each other. As seen in Figure 4b, the weight of the sample is 0.26 g, the weight of the clamp and wire is 2.42 g, and the weldment can bear more than 700 times its own weight, indicating that the material has excellent welding performance.

In the previous calculation, we obtained T_v_ = 57 °C. When the temperature is greater than T_v_, the shape of the material can be changed and processed into the target form. At the same time, due to the existence of reversible covalent bonds in the network topology, the material will be endowed with the property of shape memory. As seen in Video S1 (see Supplementary Materials), first, the spline was heated with a hair dryer for 10 s, changed to shape 1 and fixed with clips, and then heated in a 150 °C oven for 20 min to obtain permanent shape 1. It was heated again for 20 s, changed to temporary shape 2, then heated for 10 s, and the spline could then return to permanent shape 1. It was heated again for 20 s, changed to temporary shape 3, then heated for 10 s, and then the spline could also return to permanent shape 1.

### 3.5. Recycling Properties

**Figure 5:**
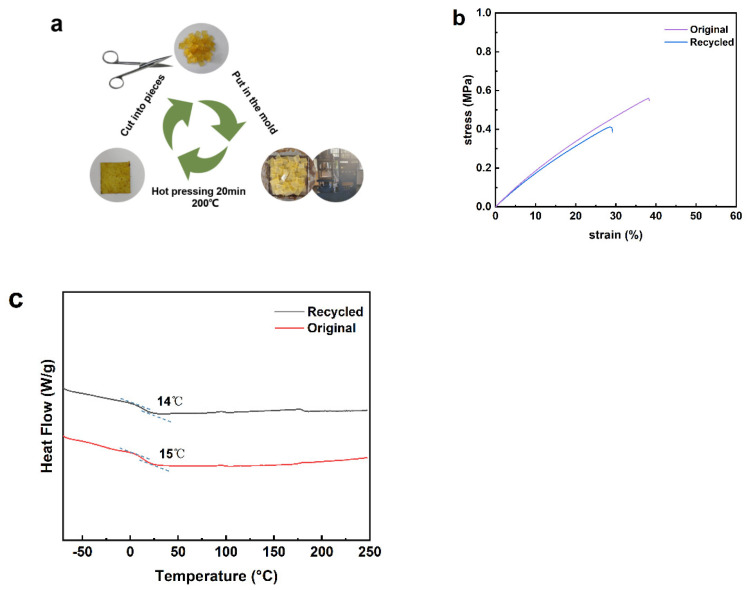
(**a**) Schematic diagram of recycling and reuse VPGE−CA_1.0_, (**b**) strain–stress curves of recycling VPGE−CA_1.0_, and (**c**) DSC curves of recycling VPGE−CA_1.0_.

Due to the network rearrangement of vitrimers at high temperatures, the VPGE−CA_1.0_ system prepared can be physically recycled at 200 °C. As shown in Figure 5a, the sample was cut into small squares of about 3 mm × 3 mm × 1 mm, and then the sample fragments were evenly spread in the flat vulcanizer mold lined with PTFE film hot−pressed at 20 MPa and 200 °C for 20 min to obtain the sample after physical recovery. As shown in Figure 5b, the mechanical properties of the recovered samples were tested, and it was found that the mechanical stress and strain at break were reduced after recovery. This may be due to the fact that there are not many dynamic structural units in the network, and the network is not tightly connected to each other, resulting in a decrease in mechanical strength. As shown in Figure 5c, DSC tests were carried out on the recovered samples, and it was found that the recovered samples were not much different from the original samples in terms of thermal performance, indicating that the recovered samples had good thermal and recyclable performance.

### 3.6. Chemical Degradability and Solvent Resistance

**Figure 6:**
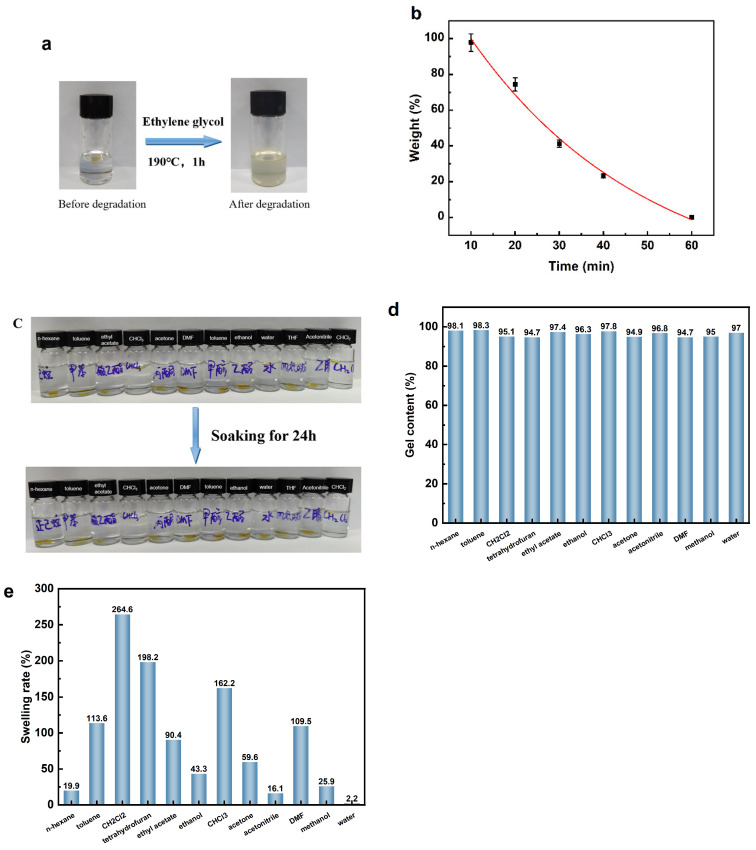
(**a**) Digital photograph of VPGE−CA_1.0_ degradation in ethylene glycol, (**b**) the curves of VPGE−CA_1.0_ degradation, (**c**) digital photograph of VPGE−CA_1.0_ soaked in different solvents, (**d**) gel content of VPGE−CA_1.0_ in different solvents, and (**e**) swelling rate of VPGE−CA_1.0_ in different solvents (see the bottle cap).

In the process of material application, in addition to physical recycling, post−processing should have requirements for solvolysis treatment in the case of damage and other conditions. VPGE−CA_1.0_ was selected to study the degradation properties of the material. The solvent was ethylene glycol, and the solvolysis experiment was carried out at 190 °C. As seen in Figure 6a,b and Video S2 (see Supplementary Materials), after 1 h of degradation, the sample was almost completely degraded. The results showed that the degradation curve was in line with the exponential decay model, which degraded rapidly within 30 min and slowed down in the next 20 min. The main reason for the degradation reaction with ethylene glycol is that the hydroxyl group contained in ethylene glycol participates in the transesterification reaction in the network under high−temperature conditions, which makes the network depolymerize and finally dissolves completely.

In order to study the solvent resistance of the material, we soaked VPGE−CA_1.0_ in different organic solvents for 24 h, then took out the sample, absorbed the excess solvent with filter paper to weigh the mass, and calculated the swelling rate according to Equation (1). The samples were dried in a 100 °C oven until they reached a constant weight, and the gel content was calculated according to Equation (2). As shown in Figure 6d, the results showed that the gel content was above 90% in different solvents, indicating that the material had excellent solvent resistance. As shown in Figure 6e, the swelling rate is different in different solvents, and the medium polar organic solvents have a higher swelling rate except for water, which may be due to the presence of ester groups and unreacted complete hydroxyl groups in the polymer structure, which may cause the sample to swell in some polar solvents with similar miscibility. The absence of swelling in water indicates that the material is hydrophobic. In addition, the rate of swelling in polar solvents also varies, and it can be seen that the rate of swelling will be large in soluble organic solvents and slightly smaller in non−soluble organic solvents.

## 4. Conclusions

In this study, we investigated the feasibility of preparing a vitrimer using the saturated product 3−pentadecylphenol derived from cashew phenol. The compound VP, containing two phenolic hydroxyl groups, was synthesized through the reaction between 3−pentadecylphenol and vanillyl alcohol. Subsequently, the two phenolic hydroxyl groups of VP were epoxidized to obtain VPGE. The curing reaction between VPGE and citric acid resulted in the preparation of fully bio−based vitrimers. The obtained sample exhibited excellent thermal performance. In the presence of the catalyst TBD, the VPGE−CA network underwent ester exchange reactions, imparting outstanding self−healing properties to the material. After repairing for 5 min at 180 °C, the cracks decreased significantly from 20 μm to 5 μm, and they nearly disappeared after 60 min. Additionally, the material demonstrated excellent welding performance, withstanding over 700 times its own weight after the welding tests. Furthermore, the vitrimers could be efficiently degraded by ethylene glycol, achieving complete degradation in just 1 h at 180 °C. Due to their excellent self−healing, recyclable, and weldable properties, the prepared vitrimers have potential applications in the field of recyclable adhesives.

## Figures and Tables

**Table 1:** The composition of various samples.

Sample	VPGE (g)	CA (g)	TBD (g)	R Epoxy/–COOH
VPGE−CA_1.0_	1.0000	0.2317	0.0252	1.0
VPGE−CA_0.8_	1.0000	0.1854	0.0201	0.8
VPGE−CA_0.6_	1.0000	0.1390	0.0151	0.6

## Data Availability

The original contributions presented in this study are included in the article/Supplementary Materials; further inquiries can be directed to the corresponding author/s.

## Supplementary Materials

The following supporting information can be downloaded at: https://www.mdpi.com/article/10.3390/polym16152113/s1, Video S1: Video for shape memory; Video S2: Video for chemical degradation.
